# Predicting group benefits in joint multiple object tracking: Predicting group benefits

**DOI:** 10.3758/s13414-023-02693-6

**Published:** 2023-06-28

**Authors:** Basil Wahn, Peter König, Alan Kingstone

**Affiliations:** 1 Institute of Educational Research, Ruhr-Universität Bochum, Bochum, Germany; 2 Department of Psychology, University of British Columbia, Vancouver, British Columbia Canada; 3 Institute of Cognitive Science, Osnabrück University, Osnabrück, Germany; 4 Department of Neurophysiology and Pathophysiology, Center of Experimental Medicine, University Medical Center Hamburg-Eppendorf, Hamburg, Germany

**Keywords:** Joint action, Coordination, Social cognition, Multiple object tracking

## Abstract

In everyday life, people often work together to accomplish a joint goal. Working together is often beneficial as it can result in a higher performance compared to working alone – a so-called “group benefit”. While several factors influencing group benefits have been investigated in a range of tasks, to date, they have not been examined collectively with an integrative statistical approach such as linear modeling. To address this gap in the literature, we investigated several factors that are highly relevant for group benefits (i.e., task feedback, information about the co-actor’s actions, the similarity in the individual performances, and personality traits) and used these factors as predictors in a linear model to predict group benefits in a joint multiple object tracking (MOT) task. In the joint MOT task, pairs of participants jointly tracked the movements of target objects among distractor objects and, depending on the experiment, either received group performance feedback, individual performance feedback, information about the group member’s performed actions, or a combination of these types of information. We found that predictors collectively account for half of the variance and make non-redundant contributions towards predicting group benefits, suggesting that they independently influence group benefits. The model also accurately predicts group benefits, suggesting that it could be used to anticipate group benefits for individuals that have not yet performed a joint task together. Given that the investigated factors are relevant for other joint tasks, our model provides a first step towards developing a more general model for predicting group benefits across several shared tasks.

## Introduction

Many tasks of daily life require people working together towards a joint goal (Sebanz et al., 2006). Common examples are preparing a meal, solving puzzles, searching people or objects, or carrying larger objects together. Often, in such tasks people tend to attain a higher performance compared to performing the same task alone – a so-called “group benefit”. To date, several studies have investigated how group benefits come about in a wide range of joint tasks, and researchers have found a number of factors that influence whether group benefits are attained (or not) and also the extent of attained group benefits (for recent reviews, see Bang and Frith (2017); Wahn et al. (2018c)). Whether group benefits are attained or not in a joint task has been shown to be highly task-dependent. That is, for joint visuospatial tasks such as collaborative visual search (for a recent review, see: Wahn and Schmitz (2022)), collective performance consistently exceeds individual performances, showing a clear group benefit. In such tasks, a prevalent coordination strategy is to quickly devise labor division strategies (e.g., each group member searches one half of the search display in a collaborative visual search task), which greatly boosts group performance. However, for shared perceptual decision-making tasks, where group members are required to negotiate a collective decision whether a certain target stimulus is present or absent, findings are more mixed. Here, several factors play a role in whether a group benefit is attained or not (for a review, see Bang and Frith (2017)). In these tasks, the prevalent coordination strategy is that group members exchange their confidence about their decision and then negotiate a mutual decision. If group members are allowed to verbally exchange their confidence about their decision, group benefits are attained (Bahrami et al., 2010). However, if only an exchange of confidence ratings is allowed, a group benefit is only attained if individual perceptual performances are similar (Bahrami et al., 2010). Regarding shared motor tasks (e.g., when jointly controlling the movements of an object towards a target location), the literature is also somewhat mixed. In some cases, a group benefit is attained (Reed et al., 2006) whereas in other cases individual performances actually exceed group performances (Knoblich and Jordan, 2003). A potential factor that has been suggested to explain these assorted results concerns the way control is distributed across group members. That is, if each group member is assigned only partial control (i.e., only one movement dimension), then no group benefits are attained. However, if both group members have control over all movement dimensions, group benefits are attained. One possible explanation that has been suggested for this difference is that in the latter case group members are free to distribute control depending on their motor capabilities, enabling them to maximize the available skills to the ultimate benefit of the group (Wahn et al., 2018b).

Regardless of the task type, two factors that have been shown to influence group performance is the availability of information about other group members’ actions and performance feedback. Regarding the former, receiving such action information has been shown to be beneficial to the group’s success in joint visual search tasks (Brennan et al., 2008; Wahn et al., 2020) and joint motor control tasks (Knoblich and Jordan, 2003) as such information can be used by group members to efficiently adapt to each other’s actions and thus facilitate interpersonal coordination. For instance, in a joint visual search task, group members, which received information on where their partner is looking were able to efficiently divide the search space and thus speed up the search (Wahn et al., 2020). Regarding the availability of performance feedback, it has been shown to be critical for attaining a group benefit in a joint multiple object track (MOT) task (Wahn et al., 2017) and to boost already-attained group benefits in a joint perceptual decision-making task (Bahrami et al., 2010) as group members use the performance feedback to calibrate the accuracy of their performed actions.

Another often investigated predictor of group performance is the degree to which its members perform similarly well when performing the same task alone (henceforth, “performance similarities”). These performance similarities are computed by dividing the individual performances by each other, resulting in a ratio indicating the degree of similarity (i.e., the closer the ratio is to 1, the higher performance similarities). This ratio is then used to predict the group’s performance when group members actually perform the same task *together*. For instance, with regard to studies investigating joint visual search, both group members in a pair perform a visual search task individually in one of the conditions. The faster individual performance is divided by the slower individual performance for each pair and the resulting ratios are correlated with the search task performances obtained when group members perform the search task together (Wahn et al., 2018a). An alternative way to compute the performance similarities is to use the individual performances measured while group members already perform a task together. This second approach is only applicable to joint tasks, where computing individual performances is feasible when the task is performed together such as in a shared perceptual decision-making task (Bahrami et al., 2010) or joint MOT task (Wahn et al., 2017). Previous research found that performance similarities positively correlated with the group benefit, such that the higher the similarity, the higher the extent of the group benefit in a visual search task (Wahn et al., 2018a). The same pattern of results has been obtained for joint perceptual decision-making (Bahrami et al., 2010) and joint motor control (Wahn et al., 2016b) task.

If similar individual performances correlate with group performance, then other interpersonal factors such as social familiarity and personality traits relevant for social interactions (e.g., empathy) may also correlate with group benefits. Indeed, previous research has found significant correlations between personality traits related to empathy, as assessed with the Interpersonal Reactivity Index (Davis et al., 1980), and group performance (Ford and Aberdein, 2015; Czeszumski et al., 2019; Koban, 2012; Ruissen et al., 2018). However, the results for familiarity are more mixed. When comparing the joint performance of strangers and friends, one study has reported a performance-enhancing effect of familiarity (Brennan and Enns, 2015a) while another has found no effects of familiarity (Ford and Aberdein, 2015).

In sum, several factors influencing group benefits have been investigated in a wide range of joint tasks and there is a clear convergence that factors such as the availability of information on the performed action of group members, performance feedback, or performance similarities are relevant to several joint tasks. To date, however, what is missing is a more integrative statistical approach that assesses these factors simultaneously rather than studies that investigate each of these factors separately. Using such an approach would be highly useful to address open questions with a high theoretical as well as practical relevance. Linear modeling would represent such an integrative approach as it could go beyond the results provided by typical statistical analyses (e.g., ANOVAs) that often consider only a few factors at once. In particular, with linear modeling we can assess how much variance factors explain collectively (**1st Modeling Objective**) and whether they are redundant or complementary in their influence on group benefits (**2nd Modeling Objective**). Addressing these points would be informative with regard to whether factors actually reflect the same underlying mechanisms (i.e., are redundant in their influence on group benefits) or reflect different mechanisms (i.e., are complementary in their influence on group benefits). From a practical perspective, linear modeling can also be used to predict group benefits for group members who have not yet performed a task together (**3rd Modeling Objective**). Addressing this point would help translate psychological research findings to more practical applications as such predictions have a high utility (e.g., they could be used to assemble more productive teams in real-life scenarios). To address these points, we opted to apply linear modeling to a joint MOT task (Wahn et al., 2017). In a joint MOT task, groups of participants are tasked with tracking multiple moving target objects among moving distractors with the aim being to maximize the number of targets tracked. This task is ideal for linear modeling as it allows one to systematically vary a wide range of factors, which have been found to be relevant for group benefits in several joint tasks (such as the availability of individual performance feedback, team performance feedback, or information about the performed actions of group members) (Wahn et al., 2018c, b). Also, the coordination mechanisms in the joint MOT task are well understood (Wahn et al., 2017, 2021). From past studies we know that group members devise efficient labor division strategies within only a few trials, and that these divisions are very frequently of a left-right nature (Wahn et al., 2017) such that group members use the midpoint of the screen as an external reference to divide the labor into left targets (tracked by one co-actor) and right targets (tracked by the other co-actor). Given that the coordination mechanisms are well understood, we are free to focus solely on assessing the modeling objectives outlined above regarding group benefits.

In the present study, we thus apply a linear modeling approach on our data from an extensive, eight experiment study, using a between-subjects design to examine the abovementioned factors’ impact on group benefits in a joint MOT task (Wahn et al., 2021). In particular, we assessed 1) how much variance factors explain collectively (**1st Modeling Objective**), 2) to what extent they are redundant or complementary in their explanatory power (**2nd Modeling Objective**), and 3) investigate the performance of the model in predicting group benefits on a separate test data set, which was not used when fitting the model, to assess the model’s potential to anticipate future group benefits (**3rd Modeling Objective**).

## Materials and methods

### Participants

In an eight experiment between-subjects design we collected data for 256 students (184 females, 72 males, *M* $=$ 21.68 years, *SD* $=$ 3.55 years) at Osnabrück University and at the University of British Columbia. Thirty-two students, grouped in 16 pairs, participated in one of the experiments. Experiments 1, 3, 4, and 8 were conducted Osnabrück University and Experiments 2, 5, 6, and 7 were conducted at University of British Columbia. We chose a sample size of 16 pairs for each experiment to equate the sample size to the sample sizes used in our previous studies on joint MOT tasks (Wahn et al., 2017; Wahn and Kingstone, 2020). We obtained written consent from all participants, and they received credits or money for their participation.

### Experimental setup

Each participant of a pair sat in front of a separate 24” monitor (screen resolution 1920 x 1020, refresh rate: 60 Hz) at a distance of 90 cm, wearing earmuffs. Each participant had a separate keyboard and computer mouse within easy reach. Participants sat at least 1.5 m apart from each other and the monitor of each participant was concealed from the other participant by a divider.

**Fig. 1:**
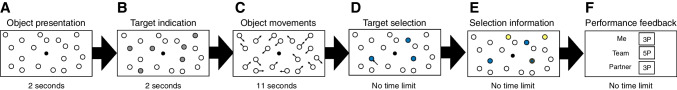
Example trial from the perspective of one participant. **A** Object presentation: 19 white objects in randomly selected positions were shown for 2 s. **B** Target indication: Six target objects turned gray for 2 s. **C** Object Movements: Objects reverted to all look white again and commenced moving in random directions for 11 s. **D** Target selection: Participants selected the objects that they thought were the targets with the computer mouse. Participants were allowed to select as many objects as they wanted and confirmed their selections by clicking on the central dot. There was no time limit. **E** Selection information: Participants saw the selections by the other group members. Overlapping selections were shown in both colors. **F** Performance feedback: Participants saw individual performance scores of themselves (Me), the other group member (Partner), and the team performance score (Team). Note, presentations in E and F had no time limit and participants could move to the next screen by pressing space. Presentations for all experiments are the same for A–D but differ for parts E and F. In particular, in Experiment 1 (No Information), participants did not see the information given in E and F. In Experiment 2 (Individual Scores), participants only saw the individual scores given in F. In Experiment 3 (Team Score), participants only saw the team score given in F. In Experiment 4 (Selections), participants only saw the selection information given in E. In Experiment 5 (Team Score + Individual Scores), participants only saw the performance feedback given in F. In Experiment 6, participants saw the selection information given in E and only the individual scores given in F. in Experiment 7, participants saw the selection information given in E and only the team score given in F. In Experiment 8, participants saw all types of information (E & F)

### Experimental procedure

Across our eight experiments, we systematically varied the availability of individual performance feedback, team performance feedback, and information about the other pair member’s target selections in a joint MOT task. Other than these types of information, there was no other way to exchange information between group members when they performed the MOT task together. Participants were not allowed to verbally communicate and could not see each other. The procedure for all experiments was the same except for the information that was received by the participants at the end of each trial.

In each trial, both participants were shown 19 white objects (0.56 visual degrees radius) in randomly selected positions for 2  s. Six objects (henceforth, referred to as “targets”) then turned gray for 2 s. Objects then reverted to white again and commenced moving in a randomly chosen fixed direction. The movement speed of each object was randomly chosen and varied between 0.90 and 1.21 visual degrees per second. While moving, objects repelled each other or the screen border in a physically plausible way (i.e., the angle of incidence was the same as the angle of reflection). Participants were required to track the movements of the targets. After 11 s, the objects stopped moving. Up to this point, both participants would see the same stimuli. Each participant was then required to independently select the objects that they thought were the targets with the computer mouse (i.e., the other group member could not see the partner’s selections). Participants were allowed to select as many objects as they wanted. After both participants confirmed their selections, participants would then either receive no information (Experiment 1), individual performance scores (Experiment 2), the team score (Experiment 3), selection information (i.e., which objects were chosen by the other group member; Experiment 4), both the individual scores and team score (Experiment 5), the selection information and individual scores (Experiment 6), the selection information and the team score (Experiment 7), or all types of information (individual scores, team score, and selection information; Experiment 8; see Fig. 1 for an example trial). A video of example trials can be accessed here: https://osf.io/q2m7h/?view_only=07f8024f18c94c61a3d6e904ec175e20

Prior to the experiment, participants were first verbally instructed by the experimenter about the task procedure and were shown an example trial. Participants were also informed that each correct selection would add one point to their individual performance and each incorrect selection would deduct one point from their individual performance. Moreover, participants were informed that we keep track of a team performance score that combines the respective individual performance scores. For this team performance, an overlapping correct and incorrect selection only adds or deducts one point from their team performance, respectively. To give an example, in Fig. 1 the participant and her partner each made three correct selections, resulting in three points each for both participants (see Me and Partner in E). However, given that one selection was overlapping, the team score only results in five points (see Team in E). For all experiments, participants were instructed to maximize the team score.

To make clear how the different types of received information relate to each other, we want to briefly outline the extent that the received information could be used by participants to deduce the team score if it was not available. If participants only received the selection information (Experiment 4), then in the case when both participants only had correct selections, it would be possible to deduce the team score (by adding up the points of all selections and counting overlapping selections only once). If participants received the selection information and individual scores (Experiment 6), they could combine both types of information to deduce the team score when there are no overlaps in the selections. If participants only receive the individual scores (Experiment 2), they cannot deduce the team score as they would not have the overlap information. In addition, we want to briefly clarify when participants can deduce their individual scores if they only receive the team score and selection information (Experiment 7). Here, it is again possible to deduce the individual scores if all selections are correct. In this case, participants can add the number of their own and the partner’s selections when seeing the selections information, to deduce the individual scores.

In each experiment, participants performed 100 trials, which took about 1 h to complete. All experiments were programmed in Python 2.7.3.

After the experiment was completed, participants were asked to fill out two questionnaires. The first questionnaire acquired a participant’s demographic data and asked how well they knew their partner on a five-point scale (1 $=$ “not at all”, 5 $=$ “very well”). The second was the Interpersonal Reactivity Index questionnaire by Davis et al. (1980), which assesses a multidimensional conceptualization of empathy (Davis, 1983). It has been validated in many previous studies and is one of the most widely used questionnaires assessing empathy (Pulos et al., 2004; De Corte et al., 2007). It includes four major factors (7 items rated on a five-point Likert scale per factor). These factors are: 1) Fantasy items: A tendency to identify with conceived characters (e.g., from movies or books), 2) Perspective-taking items: A tendency to adopt the view of other people, 3) Empathic concern items: A tendency to feel compassion/concern for others with negative experiences, and 4) Personal distress items: A tendency to feel discomfort/anxiety when witnessing negative experiences of others.

### Methods of data analysis

#### Dependent variables

As the dependent variables for our linear models, we will use two commonly used criteria of group benefits – the collective benefit (Bahrami et al., 2010; Wahn et al., 2018a) and the collaborative benefit (Brennan and Enns, 2015b; Wahn et al., 2017) criterion. The collective benefit criterion is used to assess whether a group benefit is attained at all, and the collaborative benefit criterion is used to assess whether a group benefit is attained due to a collaboration (i.e., group members devised a division of labor strategy in the joint MOT task). Using two criteria has been shown to be necessary for the MOT task given that a collective benefit can be attained without any collaboration (Wahn et al., 2021). That is, the mere statistical facilitation when performing a task together has been shown to be sufficient for attaining a collective benefit but not sufficient to attain a collaborative benefit (Wahn et al., 2021). In other words, if group members perform the same MOT task together but do not exchange any information (and also cannot see each other) and thus independently pick their targets in the MOT task, the overlap between target selections is still sufficiently low to attain a collective benefit.

A collective benefit is attained by a group if it outperforms its best member’s individual performance. As in our earlier studies (Wahn et al., 2017; Wahn and Kingstone, 2020), we computed this criterion by dividing the pair’s team performance by the better member’s individual performance for each trial. If this ratio is above one, a pair attained a collective benefit for a given trial. We then applied a moving average window (size 20 trials) to the data of each pair, extracted the peak value, and used this measure as our dependent variable in our linear models.

A collaborative benefit is attained by a group if it outperforms a simulated group performance of group members that do not collaborate (i.e., do not divide the labor). For each trial, we simulated this hypothetical performance by randomly distributing the correct selections of each group among the target objects. The same procedure was also done for the distractors by randomly distributing the incorrect selections of each group member among the distractor objects. We then computed the team score that would have been obtained for this random distribution of correct and incorrect selections. We repeated this procedure 1000 times and computed the average team score. The rationale here was that the overlapping selections for the targets and distractors should randomly fluctuate if co-actors do not follow a division of labor strategy (i.e., object selections of group members will not be systematically related to each other). They would only be related to each other if, for instance, one member tracks the left and the other member tracks the right targets. In earlier studies, we found that such a simulation approximates the team score of group members of a non-collaborative group accurately (Wahn et al., 2017, 2018a, 2021). As for the collective benefit, we computed ratios by dividing the actual team score for each trial by the simulated team score. A ratio above 1 would indicate that a collaborative benefit is attained. We again applied a moving average window (size 20 trials) to the data of each pair, extracted the peak value, and used this measure as our dependent variable in our linear models.

As a point of note, the collective benefit provides a lower threshold to assess whether a group benefit is attained or not for a joint MOT task. That is, groups that have attained a collaborative benefit also have attained a collective benefit. Yet, the reverse is not true as a collective benefit could be attained without collaboration (i.e., without a labor division). That is, as noted above, the mere statistical facilitation of performing the joint MOT together is sufficient to attain a collective benefit.

**Fig. 2:**
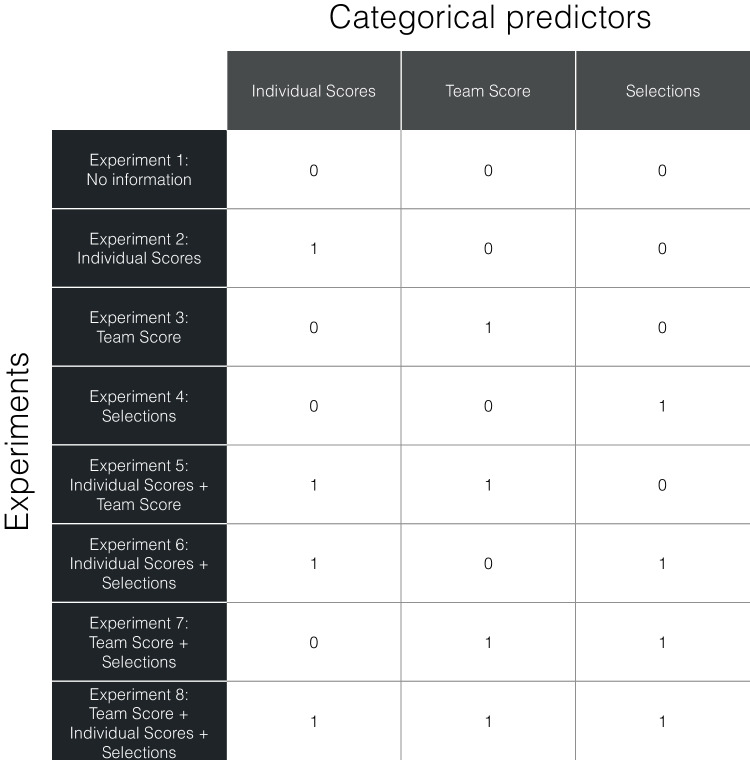
Overview how the experiments are translated into three categorical predictors for our linear regression models to predict group benefits

As in our earlier studies (Wahn et al., 2017, 2020, 2021), to assess the degree of collaboration between group members, we also calculated for each trial the fraction of overlapping selections (henceforth, referred to as “overlap”) between group members (i.e., divided the number of selections that both group members selected by the total number of selections). The lower the overlap, the better the collaboration (i.e., the better the labor division).

As noted in the Introduction, earlier studies (Bahrami et al., 2010; Wahn et al., 2016b, 2018a) have found that the similarities between individuals’ performances correlate with the extent of an attained group benefit in several joint tasks (i.e., the higher the similarities between the individual performances, the higher the collective benefit). We computed this predictor by dividing the better individual’s mean performance (across trials) by the worse individual’s mean performance (across trials) for each pair.

As a point of note, in our earlier paper (Wahn et al., 2021) we analyzed the collective and collaborative benefit ratios, the overlap, and whether group members collaborated or not. If group members collaborated, we also analyzed how they collaborated. To briefly summarize these earlier results, we found that groups can already attain a collective benefit without receiving any information (Experiment 1: No Information) or only receiving the individual scores (Experiment 2: Individual Scores). A collaborative benefit is attained if participants receive feedback about their team performance (Experiment 3: Team Score & Experiment 5: Team Score + Individual Scores) or each other’s actions (Experiment 4: Selections). In these experiments, group members devised division of labor strategies. If both types of information are received (Experiment 7: Team Score + Selections & Experiment 8: Team Score + Individual Scores + Selections), groups are faster in creating efficient labor divisions. To create labor divisions, group members used the screen center as a reference to divide the labor into a left and right side such that one group member tracks the left targets and the other group member tracks the right targets.

**Table 1:** Collective benefit step-wise regression results

	Model 1	Model 2	Model 3	Model 4
Intercept	0.00	0.00	0.00	0.00
	(0.08)	(0.07)	(0.07)	(0.05)
Individual Scores	$-0.04$	$-0.02$	0.00	0.07
	(0.08)	(0.07)	(0.07)	(0.05)
Team Score	$$0.33^{***}$$	$$0.33^{***}$$	$$0.30^{***}$$	0.02
	(0.08)	(0.07)	(0.07)	(0.06)
Selections	$$0.36^{***}$$	$$0.35^{***}$$	$$0.38^{***}$$	$$0.13^{*}$$
	(0.08)	(0.07)	(0.07)	(0.06)
Performance Similarities		$$-0.39^{***}$$	$$-0.39^{***}$$	$$-0.32^{***}$$
		(0.07)	(0.07)	(0.05)
Empathic Concern (mean)			0.03	0.02
			(0.07)	(0.05)
Emphatic Concern (difference)			$-0.11$	$-0.02$
			(0.07)	(0.06)
Personal Distress (mean)			$-0.14$	$-0.08$
			(0.07)	(0.05)
Personal Distress (difference)			$$-0.20^{**}$$	$$-0.08$$
			(0.07)	(0.06)
Overlap				$$-0.65^{***}$$
				(0.07)
R$^2$	.24	.39	.47	.71
Adj. R$^2$	.22	.37	.44	.68
Pairs	124	124	124	124

Standardized regression weights are shown for each predictor along with the standard errors in brackets, separately for each regression step

$$^{***}p<.001$$, $$^{**}p<.01$$, $$^*p<.05$$

## Results

### Collective benefits

**Fig. 3:**
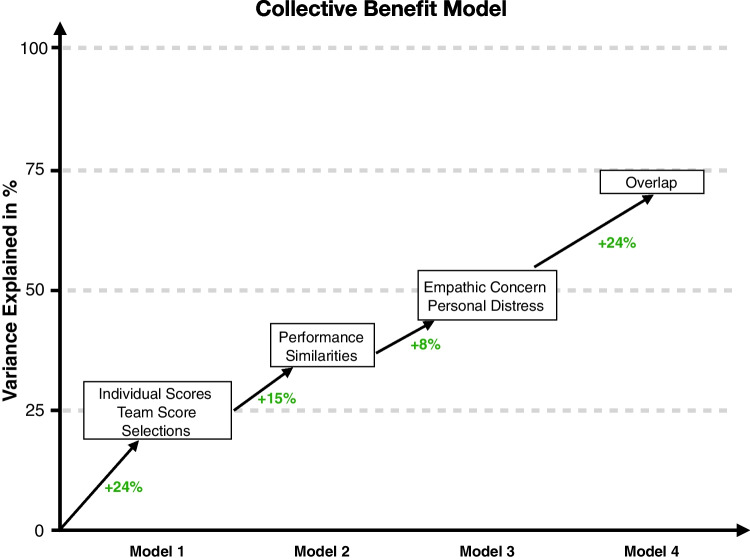
Collective benefit step-wise regression results. The variance explained (in %) is shown as a function of the models and their relative increase in explained variance (in *green*)

We first test how much variance is explained by our experimental manipulations (**1st Modeling Objective**) and assess the degree to which they are redundant or complementary in their explanatory power (**2nd Modeling Objective**). We used a step-wise multiple regression, for which we first included all factors that varied across experiments (i.e., the availability of the target selections of co-actors, the team performance feedback, and individual performance feedback) as categorical predictors (henceforth referred to as “experimental factors”). Our rationale to include them first was that direct experimental manipulations likely explain the majority of the variance (as they directly change the information that participants have available in the experiment) and all variance explained by predictors added afterwards would be controlled for the variance already explained by the experimental manipulations. The different experiments were translated into three categorial predictors for the model (see Fig. 2, for an overview). We found that the multiple regression including our experimental factors was significant (*F*(3,120) = 12.85, *p* $<.001$), explaining 24% of the variance (see Table 1 column “Model 1” for an overview and Fig. 3). Comparing the normalized regression weights of our predictors (all variables were z-scored prior to running the model), we find that only the weights for the Team Score and Selections are significant predictors and similar in size; whereas the Individual Scores predictor is not significant. These results suggest that the type of information group members receive already accounts for about one quarter of the variance and that this variance is primarily explained by the Team Score and Selections predictors.

Before adding further predictors, we briefly assessed to what extent the different data collection sites (Canada vs. Germany) explain variance. When adding this categorical predictor and performing a model comparison using an F-test, we did not find a significant effect (*F*(1,119) = 0.01, *p* $=$.905), suggesting that the data collection site had no influence on collective benefits (and thus we did not include this factor in the model). When adding all possible interaction effects between our experimental factors as predictors to the regression and running a model comparison using an F-test, we found that they did not significantly explain additional variance (*F*(4,116) = 1.71, *p* $=$.153).

Expanding our multiple regression further, in a second step, we added the performance similarities as another predictor to the model. Our rationale for including this predictor next is that it likely explains the second largest part of the variance (after our experimental manipulations) since it is based on data directly measured in the experiments and has been found to correlate with group performance in a number of tasks (Bahrami et al., 2010; Wahn et al., 2016b, 2018a). Using an F-test to compare the previous model with the new model including the performance similarities, we find that this addition is significant (*F*(1,119) $=$ 29.51 *p* <.001) and that this predictor explains an additional 15% of the variance (total: variance explained: 39%, see Table 1 column “Model 2” for an overview). These results suggest that performance similarities are also a predictor of collective benefits in the current joint task, and that this predictor explains additional variance on top of the variance explained by the other predictors already included in the model (i.e., it is not redundant to the other predictors).

Next, we assessed whether the four factors of the Interpersonal Reactivity Index questionnaire (Davis et al., 1980) and participants’ ratings how well they know each other may explain additional variance. Given that the questionnaire data was collected after the actual experiment, we included these predictors last as the data collected likely explains the least variance in the dependent variable. Prior to including the Interpersonal Reactivity Index factors, we assessed their internal consistency by computing Cronbach’s alpha for the items of each factor. We found that the computed Cronbach’s alphas are high and in a similar range (.71 –.78; Fantasy items:.75; Perspective-taking items:.78; Empathic Concern items:.76; Personal Distress Items:.71) to those obtained by Davis et al. (1980) (.68 –.79). For each set of items of each dimension, we reversed the inverted items and then added up the ratings to obtain scores for each questionnaire dimension. As a point of note, for four pairs the questionnaire data were incomplete or missing (1 in Experiment 4; 1 in Experiment 6; 2 in Experiment 8). We did not include these four pairs for all regressions to make model comparisons possible for all our analyses.

Given that our regression predicts collective benefits for *pairs* while questionnaires were filled out by each *individual* participant, we included two predictors for each questionnaire dimension of the Interpersonal Reactivity Index questionnaire to avoid a potential loss of information by averaging scores across the ratings of group members. One predictor was the mean score for each pair, and the other predictor was the absolute difference between the two scores for each pair. We added these two predictors for each questionnaire dimension separately to the model and tested for each of these additions whether they significantly explain additional variance when comparing models (again using an F-test). We found a significant effect only for the empathic concern (*F*(2,117) $=$ 3.09, *p* =.049, additional variance explained: 3%) and personal distress scores (*F*(2,117) $=$ 7.44, *p* <.001, additional variance explained: 7%).

**Table 2:** Collaborative benefit step-wise regression results

	Model 1	Model 2	Model 3	Model 4
Intercept	0.00	0.00	0.00	0.00
	(0.07)	(0.07)	(0.07)	(0.04)
Individual Scores	$-0.06$	$-0.06$	$-0.04$	0.04
	(0.07)	(0.07)	(0.07)	(0.04)
Team Score	$$0.44^{***}$$	$$0.44^{***}$$	$$0.43^{***}$$	0.07
	(0.07)	(0.07)	(0.07)	(0.05)
Selections	$$0.41^{***}$$	$$0.41^{***}$$	$$0.43^{***}$$	$$0.12^{*}$$
	(0.07)	(0.07)	(0.07)	(0.05)
Individual Scores x Team Score		0.00	$-0.03$	$-0.01$
		(0.07)	(0.07)	(0.04)
Individual Scores x Selections		$-0.02$	$-0.02$	0.03
		(0.07)	(0.07)	(0.04)
Team Score x Selections		$$-0.23^{**}$$	$$-0.22^{**}$$	$$-0.09^{*}$$
		(0.07)	(0.07)	(0.04)s
Individual Score x Team Score x Selections		0.03	0.02	0.02
		(0.07)	(0.07)	(0.04)
Personal Distress (mean)			$-0.14^{*}$	$-0.07$
			(0.07)	(0.04)
Personal Distress (difference)			$-0.16^{*}$	0.00
			(0.07)	(0.04)
Overlap				$$-0.80^{***}$$
				(0.05)
R$^2$	0.37	0.42	0.46	0.82
Adj. R$^2$	0.35	0.38	0.42	0.80
Pairs	124	124	124	124

Standardized regression weights are shown for each predictor along with the standard errors in brackets, separately for each model

$$^{***}p<.001$$, $$^{**}p<.01$$, $$^*p<.05$$

Adding both these sets of predictors simultaneously to the model resulted in an 8% increase of explained variance (total: variance explained: 47%, see Table 1 column “Model 3” for an overview). These results suggest that the empathic concern and personal distress scores explain additional non-redundant variance in collective benefits. When inspecting the regression weights for this model, the only significant predictor is the personal distress difference score predictor. Given that this regression weight is negative, our findings suggest that the lower the difference between personal distress scores, the higher the collective benefit.

Up to this point, all predictors included in the model could *in principle* be known before group members actually perform the MOT task together. Specifically, one could know in advance an individual’s solo tracking performance, their interpersonal reactivity index questionnaire results, and what type of information they will receive when they are teamed up with another participant. Thus, the current regression model could potentially be used to *predict* the extent of collective benefits of group members *before* they actually perform the MOT task together (**3rd Modeling Objective**). Nevertheless, it is worth noting that in real-life scenarios one may not know all the relevant individual measures in advance.

To test how well our regression model can predict collective benefits, or, in other words, how well it generalizes to unseen data, we performed a cross-validation approach. We repeatedly fitted the model with one pair left out of the dataset and used the fitted regression weights to predict the extent of the collective benefit of the excluded pair. To assess the prediction error, we computed the root-mean-square error (RMSE) for each excluded pair. As a point of note, standard deviations is the unit of this error measure as all the data are z-scored. We find a RMSE of 0.7897 for our cross validated data, which is an error increase of only 7.92% compared to the RMSE of 0.7221, which we obtain if the model fitting procedure includes all pairs. These results suggest that the model generalizes well to unseen data.

To assess to what extent our model might have been prone to overfitting, we repeatedly fitted our model again using a ridge regression (instead of a multiple regression) and systematically varied its Lambda parameter (which controls the degree of L2 regularization) from 0.01 to 100. We found that a Lambda of 0.1 resulted in the best model fit. We then repeated the cross-validation approach above again with the ridge regression using this Lambda value. We found that the RMSE is only marginally better than without applying the regularization (RMSE ridge regression: 0.7842 vs. RMSE multiple regression: 0.7897), suggesting that our multiple regression model did not overfit the data.

**Fig. 4:**
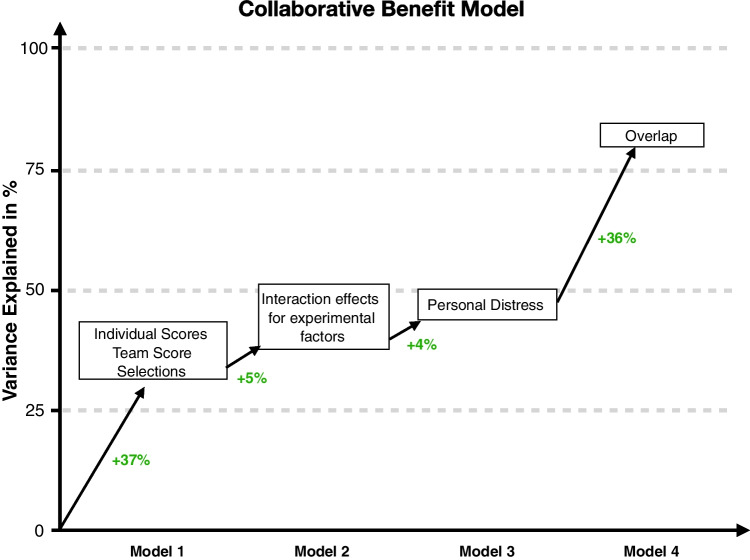
Collaborative benefit step-wise regression results. The variance explained (in %) is shown as a function of the models and their relative increase in explained variance (in *green*)

In a final modeling step, we included the overlap as a measure of the collaboration between group members as another predictor in the model. This enables us to assess the predictors in the model that are redundant with regard to a direct measure of the collaboration in the MOT task (i.e., how well co-actors divided the labor) (**2nd Modeling Objective**). We find that the overlap explains an additional 24% of the variance in the data (*F*(1,114) $=$ 90.55, *p* <.001) (total: variance explained: 71%, see Table 1 column “Model 4” for an overview). We also observe that the regression weights of our experimental factors are considerably reduced after adding the overlap predictor, suggesting that these predictors are redundant with regard to a direct measure of the collaboration between co-actors.

To summarize, our multiple regression model including several predictors of collective benefits explains about half of the variance in the data (**1st Modeling Objective**) and contains non-redundant predictors (**2nd Modeling Objective**). Moreover, the model generalizes well and could be used to also accurately predict future collective benefits (**3rd Modeling Objective**).

### Collaborative benefits

We repeated the above regression analyses to assess how the contributions of our predictors anticipate collaborative benefits compared to collective benefits. We again first included only our experimental factors and again find a significant effect (*F*(3,120) $=$ 23.06, *p* <.001). Specifically, the first model explains 37% of the variance, which is 13% more than the 24% explained in the initial collective benefit model. Comparing the normalized regression weights of our predictors (see Table 2 column “Model 1” for an overview and Fig. 4), we find that the team score and selection predictors have high weights and are both significant. Before adding further predictors, we again briefly assessed to what extent the different data collection sites (Canada vs. Germany) explain variance. We again did not find a significant effect (*F*(1,119) = 0.01, *p* $=$.941), suggesting that the data collection site also had no influence on collaborative benefits. When adding all possible interaction effects between these factors as predictors to the model, we find that these additions are significant (*F*(4,116) $=$ 2.57, *p* $=$.04). When extending this model with the performance similarities predictor, we find that this addition is not significant (*F*(1,115) $=$ 0.77, *p* $=$.38). With regard to the predictors from our questionnaire, we only find that adding the personal distress scores results in a significant addition (*F*(2,118) $=$ 4.65, *p* $=$.01; all other *ps* >.11).

We performed the same cross-validation approach as above also for this model including the categorical predictors (and their interactions) and the personal distress predictors. To assess the prediction error, we again computed the RMSE for each left out pair. We found a RMSE of 0.7890, which is an increase of 7.22% compared to the RMSE of 0.7320 obtained if the model includes all pairs. These results suggest that this model also generalizes well to unseen data (i.e., could potentially be used to predict future collaborative benefits).

As above, to assess to what extent our model might have been prone to overfitting, we repeatedly fitted our model using a ridge regression, again varying the Lambda parameter between 0.01 and 100. We found that a Lambda of 0.16 resulted in the best model fit. Repeating the cross-validation approach with the ridge regression using this Lambda value, we again only found a minor reduction in the RMSE relative to the multiple regression (RMSE ridge regression: 0.7841 vs. RMSE multiple regression: 0.7890), suggesting that our multiple regression model did not overfit the data also for the collaborative benefit model.

In a final modeling step, we again added the overlap measure as a predictor to the model, and found that this addition is significant (*F*(1,113) $=$ 220.16, *p* <.001), explaining an additional 36% of the variance (total: variance explained: 81%, see Table 2 column “Model 2” for an overview). We also found that the regression weights of our experimental factors are reduced, suggesting that they are redundant with respect to the added overlap predictor.

Overall, we find a similar pattern of results as above when predicting the collective benefits. Again, our experimental factors make major contributions towards explaining variance also for the collaborative benefit (**1st Modeling Objective**). When expanding the model, the performance similarities predictor is now redundant with regard to our experimental factors. The personal distress questionnaire predictor, however, still explains additional variance (**2nd Modeling Objective**). Again, the model generalizes well to unseen data (**3rd Modeling Objective**).

## Discussion

In this paper, we addressed three modeling objectives. We assessed 1) how much variance factors explain collectively with regard to group benefits (**1st Modeling Objective**), 2) to what extent they are redundant or complementary in their explanatory power (**2nd Modeling Objective**), and 3) the performance of the model in predicting group benefits on a separate test data set, which was not used during model fit, to assess the model’s potential to predict future group benefits (**3rd Modeling Objective**).

For the first and second objective, we find that several predictors (i.e., our experimental factors, performance similarities, questionnaire data) collectively explain half of the variance (**1st Modeling Objective**) and make non-redundant contributions towards predicting group benefits (**2nd Modeling Objective**). Also, when our regression models only included predictors that could be measured *prior* to co-actors performing the MOT task together, we found using a leave-one-out cross-validation approach that our models accurately predict future group benefits (**3rd Modeling Objective**). In the following, we discuss each of our modeling findings in more detail and their contributions to the literature.

In our models, we first added the experimental factors alone (i.e., the different types of information received by the group members depending on the experiment) and found that they already explained sizeable portions of the variance, with significant contributions from both selection information and team performance feedback predictors. These findings suggest that the availability of these types of information influence group benefits, supporting the view that they are particularly important for group benefits in joint spatial tasks (Brennan et al., 2008; Wahn et al., 2020). When comparing the weights of these predictors, we also extend earlier results by finding that they are comparable in magnitude, suggesting that these sources of information are equally important with regard to group benefits. Moreover, earlier findings are also extended as we show that the availability of individual performance feedback does not significantly contribute towards predicting group benefits, suggesting that feedback about one’s own performance is not critical information for group members for improving group performance. Future studies could assess whether team feedback and information about the performed actions of co-actors also contributes equally towards predicting group benefits in other joint tasks or whether one of these types of information has a higher weight. For instance, in joint motor tasks, it could be that information about the co-actor’s actions is more important because the primary coordination mechanism here is that co-actors adapt their performed actions to each other to optimize group performance (Wahn et al., 2018b). Also, given that we base our conclusions on comparing the magnitude of regression weights, future studies could also use more direct experimental manipulations (e.g., a within-subjects design, in which different types of the received information are compared) to confirm the relative importance of these different types of information.

When extending the models with the performance similarities predictor, we found that this predictor significantly explains additional variance. These findings extend earlier results (Bahrami et al., 2010; Wahn et al., 2016b, 2018a) as they indicate that this predictor accounts for complementary variance relative to the performance feedback and selection information. We thus suggest that it likely accounts for variance in group benefits related to the individual performance capabilities, which are independent of the co-actors’ abilities to collaborate with each other. In terms of applications, these findings suggest that a first step towards assembling an effective team could be to choose team members that perform the task that will be performed jointly similarly well alone. Adding the performance similarities predictor to the collaborative benefit model, however, did not significantly increase the explained variance. To explain this difference in our results between our two models, we suggest that the simulated independent performance used to assess collaborative benefits may already account for the similarities in the individual performances. That is, given that the simulated independent performance is based on repeatedly sampling trials from the individual selections of both group members, differences in the individual performances were likely already incorporated when assessing collaborative benefits. In addition, given that this finding is based on a correlation, future studies would be needed that compare the joint performance of for two conditions with pre-selected participants based on their individual performances to show a direct influence of performance similarities on group benefits. That is, one condition would be composed of pairs with group members that have similar individual performances whereas the other condition would be composed of pairs with group members with dissimilar individual performances. Comparing these conditions could then confirm whether the individual performance similarities in fact do have a direct influence on group benefits.

When further extending our models by adding predictors from our empathy questionnaire (Davis et al., 1980) to our models, we found that adding personal distress scores led to significant increases in the explained variance, indicating that this predictor also explains additional non-redundant variance. These personal distress scores have been interpreted as a measure of feeling discomfort or anxiety when witnessing negative experiences of others (Davis et al., 1980). When interpreting the regression weights, our findings suggest that the more similar personal distress scores are, the higher the group benefit. Our findings thus indicate that members with more similar personal distress scores may more easily converge on an effective collaboration strategy, resulting in higher group benefits. Yet, we acknowledge that this interpretation is speculative and given that we had no concrete hypothesis or prediction with regard to our questionnaire predictors, we suggest that further studies are needed to confirm whether personal distress scores reliably contribute towards predicting group benefits.

After adding the predictors described above, we also tested how well the models generalize to data that is not part of the model fitting procedure. That is, we tested how well the models would predict group benefits for group members prior to performing the MOT task together. We found that the prediction error was only marginally increased for data that was not part of the fitting procedure. Thus, the collective and collaborative benefit models have the potential to also predict future group benefits. These findings suggest that such models may prove to have a high utility as they could also be used to assemble individuals in teams that would be predicted to perform well together. As such, we extend earlier findings as previous studies did not assess the predictive potential of factors relevant to group benefit. We show that these factors not only explain sizeable portions of the variance of group benefits in the joint MOT task but that they also could be used to predict group benefits. However, to confirm the utility of such a model, it is important that a future study tests whether pairs composed of pre-selected group members based on the model actually do perform close to the predicted group benefits.

In a final modeling step, we also included the overlap measure – a direct measure of the collaboration between group members in the MOT task – to assess which predictors are redundant with regard to this variable. We found that the regression weights of our categorical predictors representing our different experimental manipulations were considerably reduced, suggesting that these factors are redundant with regard to the overlap measure, and thus directly related to co-actors’ collaborative behavior. These results are relevant with regard to devising a model that can predict future group benefits. In particular, these results suggest that a direct measure of the actual collaboration between group members can at least in part be replaced by predictors indicating which information group members will receive when working together. In other words, one can predict, to a degree, how well the group members will collaborate based on the information that the group members will receive. However, these findings are of course limited to the current joint MOT task and future studies investigating other joint tasks are required to test whether findings generalize to other tasks or not.

While the present work has considered the contributions of several factors towards predicting group benefits, there are several other factors left to explore to further refine predictive models. For instance, it is unclear to what extent social facilitation and impairment effects explain variance in group benefits. That is, earlier work has shown that the mere presence of others can lead to increases in arousal which can lead to improvements or reductions in individual performance (for a review, see Belletier et al. (2019)). Such changes in individual performances could in turn affect the extent of group benefits. However, what is unclear is how changes in arousal would influence group members’ willingness to collaborate and the efficiency of collaboration (e.g., how well they divide the labor). In a similar vein, the increase in arousal due the presence of others can also lead to unintentional imitative tendencies (Belot et al., 2013; Cook et al., 2012; Doyen et al., 2012; Naber et al., 2013). Future studies could address to what extent these imitative tendencies affect interpersonal coordination and thus group benefits. Other potential factors to explore could be physiological measures relevant to attentional selection such as event-related potentials (Drew et al., 2009; Sternshein et al., 2011) or pupil size (Wahn et al., 2016a) as a correlate of attentional effort. These measures may explain additional variance in group benefits, which are not captured by other predictors, which are all behavioral measures.

Finally, a critical and open question for future investigations is whether the same or a similar set of predictors as used in the present study would also predict group benefits in other joint tasks. We have already touched on this question in a different study when we applied a regression model with a similar set of predictors to a joint visual search task and found comparable results (Wahn et al., 2020). This suggests that at least for spatial joint tasks a similar set of predictors is highly relevant for predicting group benefits. With regard to other tasks, previous research on joint motor control tasks found that performance similarities are a predictor of group benefits (Wahn et al., 2016b) and that the availability of information about the co-actor’s performed actions is highly important for coordination (Knoblich and Jordan, 2003). These findings suggest that a similar model as used in the present study may also predict to a degree group benefits in joint motor control tasks (for a recent review on group benefits in joint motor tasks, see Wahn et al. (2018b)). Apart from joint motor control tasks, the present set of predictors could also be applied, for instance, to shared memory tasks. In joint memory tasks (for general reviews, see Bietti and Sutton (2015); Rajaram and Pereira-Pasarin (2010)), co-actors need to memorize a list of words. There are a number of clear similarities between such a task setting and the present joint MOT task. For instance, co-actors may divide the labor by having each co-actor memorize a complementary subset of words and also similarities in the individual abilities to memorize words may influence the extent of group benefits. Ultimately, future studies that would test whether the set of predictors used in the present study also predicts group benefits in other joint tasks could (in the long term) lead to constructing a more general model for the prediction of group benefits across many different types of joint tasks. This would not only enable researchers to extract commonalities and differences in the factors, which are relevant to group benefits for different joint tasks, but also help to predict future group benefits using one general model for several joint tasks.

### Open practices statement

The study has not been pre-registered. All data and a video of example trials can be accessed in the following repository: https://osf.io/q2m7h/?view_only=07f8024f18c94c61a3d6e904ec175e20. Experimental code and analysis scripts are available on request.
